# Circulating Tumor Cells Adhesion: Application in Biosensors

**DOI:** 10.3390/bios13090882

**Published:** 2023-09-12

**Authors:** Eduarda B. Paglia, Estela K. K. Baldin, Gabriela P. Freitas, Thalyta S. A. Santiago, João B. M. R. Neto, Jorge V. L. Silva, Hernandes F. Carvalho, Marisa M. Beppu

**Affiliations:** 1 School of Chemical Engineering, Department of Process and Product Development, University of Campinas, Campinas 13083-852, Brazil; e203320@dac.unicamp.br (E.B.P.); estelakerstner@gmail.com (E.K.K.B.); gabrielafreitas197699@gmail.com (G.P.F.); t211758@dac.unicamp.br (T.S.A.S.); 2 Renato Archer Information Technology Center, Campinas 13069-901, Brazil; jorge.silva@cti.gov.br; 3 Technology Center, Federal University of Alagoas, Maceió 57072-900, Brazil; jbmrneto@gmail.com; 4 Institute of Biology, Department of Structural and Functional Biology, University of Campinas, Campinas 13083-864, Brazil; hern@unicamp.br

**Keywords:** electrochemical biosensor, circulating tumor cells, CD44, integrins, EpCAm

## Abstract

The early and non-invasive diagnosis of tumor diseases has been widely investigated by the scientific community focusing on the development of sensors/biomarkers that act as a way of recognizing the adhesion of circulating tumor cells (CTCs). As a challenge in this area, strategies for CTCs capture and enrichment currently require improvements in the sensors/biomarker’s selectivity. This can be achieved by understanding the biological recognition factors for different cancer cell lines and also by understanding the interaction between surface parameters and the affinity between macromolecules and the cell surface. To overcome some of these concerns, electrochemical sensors have been used as precise, fast-response, and low-cost transduction platforms for application in cytosensors. Additionally, distinct materials, geometries, and technologies have been investigated to improve the sensitivity and specificity properties of the support electrode that will transform biochemical events into electrical signals. This review identifies novel approaches regarding the application of different specific biomarkers (CD44, Integrins, and EpCAm) for capturing CTCs. These biomarkers can be applied in electrochemical biosensors as a cytodetection strategy for diagnosis of cancerous diseases.

## 1. Introduction

Cancer is a significant public health problem, being the second leading disease with highest mortality rates worldwide [1,2]. According to the International Agency for Research on Cancer (IARC), cancer cases will increase by approximately 50% between 2020 and 2040 [3]. Early detection and isolation of cancer cells is essential for understanding and treating this type of disease [4]. Circulating tumor cells (CTCs) refer to a population of cells that have detached from the tumor and are circulating in the peripheral blood and/or lymphatic system. They are found in almost all solid malignant tumors and play a crucial role in the metastatic process [5,6,7]. Furthermore, recent studies have indicated that tumor cells can disseminate even in the early stages of tumor progression [8,9,10].

In this context, the diagnosis of cancer through the capture and analysis of CTCs has become a crucial breakthrough for studying the progression and control of oncological tumors [11]; being essential both for early prognosis and for advancing and monitoring treatment [12]. Currently, the development of CTCs isolation techniques is based on comparing biophysical and biochemical properties of CTCs to the properties of blood cells. The properties used for this purpose include to size, density, deformability, electrical and magnetic properties, and expression of cellular markers [13,14]. However, such differentiation becomes limited due to their proportion in peripheral blood [15], with the presence of 1–10 CTCs estimated per billion blood cells [13].

The diagnosis through the detection of CTCs is a less invasive and more accessible liquid biopsy approach than traditional diagnoses [16]. Among these, the CellSearch system is currently the only technology approved by the FDA for determining the prognosis of patients with advanced breast, prostate, and colorectal cancer through CTC enrichment [11] expressing epithelial cell adhesion molecules (EpCAM) and cytokeratin [14,17]. However, as CTCs undergo this epithelial-mesenchymal transition (EMT), the expression of EpCAM and other epithelial markers may decrease [14], leading to lower sensitivity of the system [11]. Additionally, subsequent analysis of isolated CTCs may also be limited due to antibody labeling [7,13]. Therefore, for highly sensitive and selective detection of CTCs, there is still a technological challenge, and thus, the development of new biomarkers appears to be a promising approach in this application area [18].

Techniques based on CTCs adhesion through the expression of biomarkers can explore different strategies for modifying the adhesion surface, such as structural alterations through nano topographical features and/or the use of chemical functionalization that may or may not contain genetic ligands for biorecognition [13], such as antibodies, proteins, and aptamers [19,20] However, CTCs exhibit heterogeneous characteristics, and their presence in minute quantities makes it challenging to identify a universal biomarker for their detection and identification [21,22].

In addition to the perspective of using biomarkers, the employment of biosensor technology as a sensing platform for CTCs capture enables the use of a simple, practical, economical, and non-invasive technique [16,23]. Moreover, in the case of biosensors that apply electrochemical techniques, advantages such as high reproducibility and sensitivity are also achieved [24]. Electrochemical sensors are based on the electron transfer at the analyte-electrode interface, involving an analyte-receptor [25]; such interaction can be analyzed through different detection modes, such as potentiometric, amperometric, conductometric, impedimetric, and voltammetric measurements [26]. Thus, the prediction of the pathological stage of cancer can be obtained through electrochemical biosensors, as the tumor lineages are detectable, despite their heterogeneity, by changes in the capacitive and resistive natures of the cell [27].

Furthermore, in recent years, studies have focused on increasing the bio-specificity of functional electrodes through the immobilization of various types of cellular marker biorecognition ligands [28]. Interaction with biomarkers such as CD44 [29], integrins [30] and EpCAM [31] proved to be a promising strategy for the development of biosensors with high sensitivity and specificity. Some research has also identified the application of 3D micro/nanoelectrodes as an effective way to capture CTCs [32] or to enhance the sensitivity and reproducibility of these electrodes through surface modification using nanomaterials [33].

Therefore, this review will discuss current approaches to the different forms of biomarker immobilization, by employing surface functionalization for the capture of circulating tumor cells. Additionally, a brief discussion on the utilization of these biorecognition elements applied to electrochemical sensing platforms will be presented, with a focus on different types of functional transduction electrodes. Finally, future perspectives will be discussed regarding the application of both the aforementioned topics in the early diagnosis and/or assistance in the treatment of oncological diseases.

## 2. Biosensor

**Figure 1:**
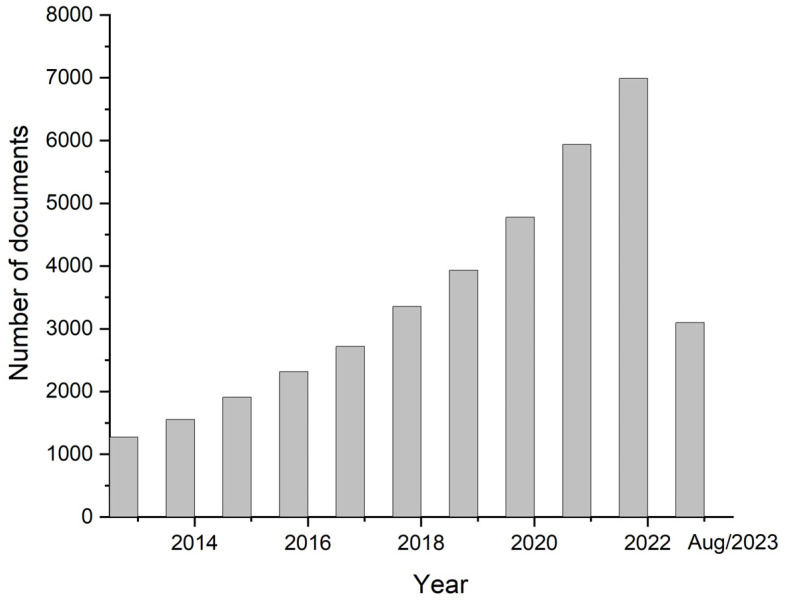
Number of publications related to the topic of this article in the last 10 years, using Scopus as a database (keywords “electrochemical sensor” and “tumoral cells”).

Biosensor devices allow the determination of relevant biomarkers by generating signals of a substance of interest, which can be used for CTCs detection. These techniques not only may be used to study the mechanism of cancer metastasis but also allow CTCs to be detected in a minimally invasive way, by a method denominated liquid biopsy [34]. Thus, there has been a great deal of interest in the development of biosensors for detecting CTCs, as indicated by the statistical data shown in Figure 1.

Biosensors have the advantages of being simple equipment and inexpensive [35]. However, a biosensor system for cancer detection poses the challenge of determining relevant biomarkers or biomarker patterns on CTC surfaces or directly on tumor tissue [36]. In the analysis of cancer biomarkers, bio-affinity-based electrochemical biosensors are usually applied to detect protein biomarkers [35].

Biosensors are formed by three components: receptor layer and/or biorecognition element, transducer, and signal evaluation module [28], with the recognition element being one of the most critical components [30]. The development of the biosensor must be specific to selectively interact with the analyte of interest, which may be a specific molecule, biomarker, or target of diagnostic importance, thus converting the resulting parameters into an accurate reading signal for the diagnosis of the disease [25].

Electrochemical sensors have excelled in the field of quantitative detection of cancer cells, including breast, prostate, liver, and cervical cancer cells [24]. These electrochemical biosensors are devices designed to analyze the behavior of an electroactive surface. They work through the interaction of a transducer electrode with the surface of interest, providing quantitative or semi-quantitative analytical information [33]. In the case of the interface with CTCs, which have different morphologies, internal molecular structures, and metabolism than normal blood cells, consequently presenting a different dielectric constant from these cells, which will always have the same constant value [24,37]. In addition, electrochemical biosensors can predict the pathological stage of cancer, since screening for differentiation and quantification of heterogeneity of tumor lineages can be measured by their distinct capacitive and resistive natures [27].

Other types of transducers in addition to electrochemical techniques can be used in cancer detection, such as optical transducers (colorimetric, fluorescent, luminescent), calorimetric transducers (thermistors), mass variation transducers (piezoelectric/acoustic waves) and magnetic transducers [27,28,30]. However, biosensors based on electrochemical techniques have significant advantages in the early diagnosis and prognosis of tumors, due to their high sensitivity and specificity, simple components, and low price [24]. One of the most classical electrochemical biosensors for tumor detection is cell impedance sensing technology, based on impedance changing at the microelectrodes interface due to growing cells on their surfaces [24]. 

### Electrodes Used in Electrochemical Sensors Aiming at CTC Adhesion

The development of electrodes for application in electrochemical transducers aimed at diagnosing tumor diseases prioritizes the search for surfaces with high catalytic activity as well as obtaining an interface with high specificity regarding cell fixation [24]. Thus, current demands are mainly focused on the investigation of different materials, changes in their surface properties, as well as the development of new technologies for manufacturing devices [38].

Materials consisting of carbon, gold, and titanium have been widely used in the manufacture of electrodes, due to their high stability, biocompatibility, and signal amplification effect [39,40,41]. In addition, the sensitivity, specificity, and efficiency of these electrodes can be improved by combining materials, such as the incorporation of graphene, metal nanoparticles, and polymeric materials [42,43,44,45]. Safavipour et al. (2020) developed electrodes based on TiO_2_/graphene oxide, observing the increase in resistance on the transfer of electric charge due to the incorporation of TiO_2_ nanoparticles [41].

Shi et al. (2016) proposed topographic changes in PDMS polymer electrodes as a promising approach for capturing CTCs, independent of surface marker expression or size of CTCs. Comparatively, nanograting structures with nanometric dimensions showed higher selectivity and efficiency for the capture of different cell lines (MCF-7, HeLa, MDA-Mb-231) based on cell adhesion by providing better contact orientation about the geometry of the nanopillars [46]. However, the existing limitation in 2D platforms in terms of the non-mimicry of the cellular environment can result in the reduction of cell adhesion, affecting the detection limit for application in cytosensing [47].

Thus, in recent years, nanostructured electrodes obtained by physical and chemical methods have been investigated due to their better surface-to-volume performance, boosting not only the conductivity but also the chemical interaction with receptor agents [38,48]. Three-dimensional (3D) and nanohybrid arrays have shown excellent performance in terms of increasing surface area exposure [49]. Xu et al. (2015) demonstrated that carbon nanotubes functionalized with indium tin oxide significantly improved the sensitivity of the electrochemical detection method, presenting a wide linear range when used as electrochemical transducers [50].

Damiati et al. (2018) developed electrodes composed of multiwall carbon nanotubes, functionalized with chitosan, with high sensitivity to the synergistic effects promoted by the superficial adhesion of tumor cells and high specificity for the detection of liver cancer cells concerning mammary cells [51]. In 2019, Wang et al. investigated electrodes in the form of gold nanostars with a diameter of 60 nm, uniformly dispersed on a carbon platform, as a support for specific aptamers of CTCs. Due to molecular recognition of the aptamer combined with reduced resistance to electron exchange by the presence of gold, the authors reached a detection limit of 5 cells·mL^−1^ for for the MCF-7 line as well as a specificity of CTCS for normal cells in samples of blood [40]. In 2021, Chen et al., proposed electrodes formed by polystyrene microtubes on a mesoporous silica structure for use in an electrochemical cytosensor, aiming at capturing MCF-7 cells line. The authors obtained a linear detection range of 1.0 × 10^7^ cells·mL^−1^ and a detection limit of 4 cells·mL^−1^, denoting excellent electrochemical and selectivity behavior for the proposed platform [47]. Wang et al. (2021) developed an electrochemical biosensor composed of a vertical tetrahedral DNA structure, used to modify a screen-printed gold electrode, and an inverted tetrahedral DNA structure multivalently bonded with aptamers. They achieved a linear range of 1 to 10^5^ MCF-7 cells with a detection limit of 1 CTC [52].

On the other hand, the advancement of technology related to manufacturing techniques is allowing the development of three-dimensional platforms for obtaining standardized functional electrodes with complete, sensitive, and low-cost geometries for diagnostic purposes [53]. In 2018, Hamzah et al. presented a mini-review showing potential conductive materials to be used in electrochemical sensors obtained using additive manufacturing [54]. The authors conclude that despite the technology presented showing the development of robust and precise electrodes, there is still a need for appropriate analytical comparison with other conventional methods.

Recently, Rocha Neto et al. (2022) presented a review focused on the development of 3D electrodes obtained by additive manufacturing for tumor cell detection. The authors presented the main advantages and advances of three-dimensional printing for the diagnosis of tumor diseases, highlighting that the development of complex geometries can be one of the ways to improve the properties of selectivity and sensitivity compared to conventional detection methods [32].

**Figure 2:**
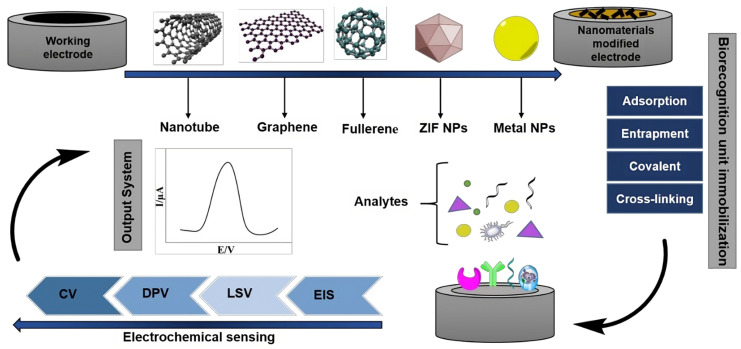
A potential approach involves creating label-free electrochemical biosensors for cancer detection. This entails enhancing electrodes with nanotechnology-based materials, immobilizing biorecognition elements using specific protocols, and utilizing robust electrochemical detection methods [26].

Figure 2 exemplifies several strategies aimed at creating electrodes to be used as platforms for electrochemical transduction in biosensors for the detection of oncological diseases. These approaches emphasize the importance of advancing electrodes constructed from nanotechnological materials, as well as the immobilization of biological recognition components of circulating tumor cells. Finally, together with the development of nanostructured electrodes to act as transduction elements, understanding the biological recognition rules for different CTC lineages requires a complex investigation of the expression of biomarkers, as well as their interaction with the support electrode that will transform biochemical events into electrical signals in electrochemical cytosensors.

## 3. Approaches for CTCs Adhesion

**Figure 3:**
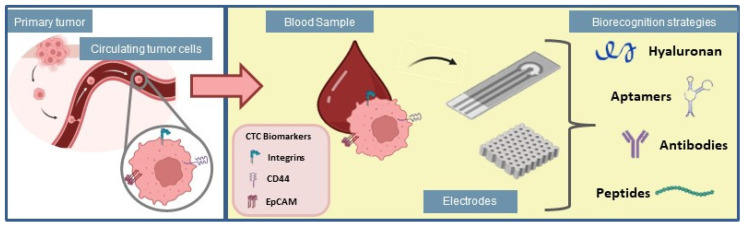
Biorecognition strategies for application in electrochemical biosensors used for adhesion of circulating tumor cells.

The number of CTCs is commonly used as a marker for cancer progression, even at early stages, to predict tumor survival [55,56], which suggests that the detection of CTCs represents a label-free strategy for cancer diagnosis and clinical management. Multiple strategies have been used to detect CTCs, however, these cells are extremely rare and mixed with normal blood components, which requires technological approaches able of isolating and selectively detecting them [57]. Hence, the study of new and rapid methods for CTCs detection is essential for timely cancer diagnosis. In this section, different strategies for selective capture of CTCs are described, highlighting their most recent applications in cancer diagnosis, and therapy. Such interactions between different biorecognition strategies and electrochemical cytosensing platforms are illustrated in Figure 3.

### 3.1. CD44

CD44 is a polymorphic glycoprotein broadly distributed in different isoforms on the surface of a wide variety of cells [58]. Due to its large variety of isoforms, this glycoprotein plays different roles in cell behavior, being involved in different cell functions such as cell adhesion, sensing, and signaling [59]. Despite its presence on non-tumor cells, CD44 is commonly explored as a cancer-related biomarker since it is overexpressed on the cell surfaces of major cancers, such as pancreatic [60], breast [61], prostate [52], lung [62], and gastric cancer [63].

As a biomarker for cancer, the level of CD44 present in the tumor cells has an essential role in cancer incursion, evolution, and metastasis. Several techniques are traditionally used for CD44 antigen detection, such as imaging [64], flow cytometry [65] magnetic resonance [66], enzyme-linked immunosorbent assay (ELISA), and other labeled methods [67]. However, these techniques tend to be time-consuming procedures, expensive and have poor performance in terms of limit of detection [68], which motivates the development of low-cost, fast, and user-friendly methods for the detection of CD44 antigen.

Monitoring CD44 on CTCs in terms of detection and quantification can provide a significant improvement in clinical cancer diagnosis. Thus, a large number of platforms with sensitive and specific properties, especially biosensors, have been developed to detect selectively CD44 aiming at cancer diagnosis applications and CTCs detection [68]. The main ligand for CD44 is hyaluronic acid (HA), an abundant component of the extracellular matrix (ECM) expressed by stromal and cancer cells [69,70]. The interaction between CD44 and HA is mediated by hydrogen bonds [71] and it occurs in the N-terminal hyaluronan binding domain (HABD) present in CD44 [71,72].

Several platforms for CD44 detection rely on the interaction of HA with CD44. Indeed, this specific interaction has been widely explored as a strategy to produce biomaterials able to detect CD44 overexpressed tumor cells. In this scenario, nanomaterials represent versatile applications in developing cancer diagnosis approaches. Using hyaluronidase and anti-CD44 antibody, Rocha Neto and colleagues concluded that the availability of CD44 receptors and the level of HA are key factors to modulate the adhesion mechanism of prostatic tumor cells on HA-based nanofilms [73]. These nanofilms were also used to functionalize interdigitated electrodes for the detection of prostatic tumor cells by using electrical impedance spectroscopy, distinguishing them in the range from 50 to 600 cells·µL^−1^ in vitro experiments [74].

Khang and co-workers reported the development of a label-free electrochemical sensor using the ligand-protein interaction for CD44 detection. The authors conjugated HA into carbon nanotube composites to capture CD44 selectively in human serum and cancer cells. The sensor demonstrated high selectivity and reproducibility with a detection limit for direct sensing of 5.94 pg·mL^−1^ without any post-labeling for amplification [75]. Amorim and co-workers presented an LbL system using HA and PLL to study the substrates’ interactions with CD44 in two human gastric cancer cell lines that overexpress this receptor (AGS and MKN45). The authors considered the influence of different HA molecular weights (6.4, 752, and 1500 kDa) and two different film interactions: covalent interaction (HA cross-linking) and electrostatic interaction. The authors presented the non-covalent interactions had limited stability compared to covalent interactions [70].

Li and co-workers utilized the multifunctional nanoprobe based on the HA-CD44 interaction for image-guided photothermal therapy in human breast carcinoma cells (MCF-7 line). The authors used the CD44-HA interaction to target the cancer cells and thiolated-hyaluronic acid labeled with Nile blue was used to stabilize the nanoprobes. They concluded that the bioprobes fabricated can be excellent candidates to realize rapidly due to the precise image photothermal therapy obtained [76].

Jeong and co-workers presented a fluorescence-sensing platform for CD44 detection. The platform was based on gold-coated graphene oxide hybrid material (GO/AuNPs) with CD44 aptamer to interact with the HA binding domain. The authors compared the GO/AuNPs with GO alone, and the former presented higher sensitivity and specificity for CD44 detection. The authors concluded that the fluorescence sensing platform used can be developed for various target molecules based on their specificity, sensitivity, and simple method [77].

Liu and co-workers proposed an electrochemical cytosensor to detect HeLa cells based on the overexpression of CD44 in these tumor cells. The cytosensor was based on the interaction between HA-CD44 and HA and was grafted into a 3D multi-walled carbon nanotube. The 3D structure improved the surface area, increasing the amount s of HA. The cytosensor presented a detection limit of 70 cells·mL^−1^ with higher selectivity and sensitivity [78].

Using a strategy without the interaction of HA-CD44, Paltusheva and colleagues reported the development of a zinc oxide fiber-optic biosensor for the detection of CD44. The biosensor was also tested with a control PSA protein and without CD44 antibodies proving to be sensitive to CD44 detection with a detection limit of 0.8 fM [79]. Kumar and co-workers developed an electrochemical biosensor to detect CD44 in breast cancer based on graphene quantum dots. The authors reported a selective and sensitive detection with a linear response in the range between 1.0 pg·mL^−1^ and 100.0 ng·mL^−1^ in spiked serum samples. Just like HA-based biosensors, both reported platforms could also be used to detect CD44, making them suitable biosensors to detect CD44 biomarkers in cancer diagnostics [29].

### 3.2. Integrins

Integrins are another cancer targeting related to tumor progression and metastasis. They are composed of two non-covalently subunits designated “α” and “β” [80]. Integrin deregulation contributes significantly to several pathophysiological states, such as deleterious embryonic development, autoimmune diseases, cardiovascular diseases, thrombosis, and cancer [81,82].

The alteration of integrins function is correlated with a range of steps in tumor progression and metastasis, such as invasion of the extracellular membrane, detachment of tumor cells from the primary site, and cell spread in the circulation and attaching to target organs promoting secondary lesions [83]. The correlation of integrins with tumor progression is an opportunity to improve cancer diagnostics and directed therapies [84]. Some integrins, such as αvβ3, α5β1, and αvβ6, are usually expressed at low or undetectable levels in most adult epithelia but can be highly upregulated in some tumors [85].

Integrin αvβ3 is overexpressed in different tumor cells, and its expression has been linked to invasiveness and metastatic potential of malignant tumors [86]. An increase in the expression of activated αvβ3 receptors has been reported to be correlated with metastasis to the bone in prostate cancer [87,88], breast cancer [89], lung cancer [90], and glioblastoma [91].

The integrin ligands to the Arg-Gly-Asp (RGD), as αvβ1, αvβ3, αvβ5, αvβ6, αvβ8, and α5β1, recognize the motif RGD activating a range of intracellular signaling pathways [92]. RGD motif is the most effective short peptide sequence for stimulated cell adhesion to surfaces [93,94]. The mechanism of RGD motif recognition and subsequent cell attachment is based on integrins on cell surfaces [95], which are promising cancer targets. Thus, these peptides may be associated with biomaterials for promoting specific cell adhesion [92,96,97].

The immobilization of RGD in a biomaterial is considered an approach to enhancing cell adhesion due to the advantages of its applications, the RGD functionality is kept after the protein biosynthesis process, and the process is considered cheap, which is a benefit for clinical application, and finally, the motif RGD can be attached to materials surfaces with variables orientations and densities [97]. In this context, Jiant et al. (2017) present the possibility of using polyethylene glycol hydrogels functionalized with Cage-RGD peptide for the screening of PC-3 tumor cells, based on high-throughput microarrays, these occur through the release of RGD motif from Cage-RGD when PSA protease is secreted from PC-3 [98].

Artificially engineered proteins with designed targets can be used as a strategy to bind biological systems. Flora and co-workers synthesized elastin-like polypeptides (ELP) containing RGD motif to adhere to HUVEC and HFF1 cells [99]. The findings indicated that the inclusion of bioactive sequences within the recombiners facilitated the replication of ligand-like properties, enabling interactions upon grafting onto a solid substrate. This approach provides a versatile and effective solution for addressing diverse biological and engineering challenges that necessitate precise control over the spatial and temporal arrangement of cells.

To study the development of nanoparticles with multiple functions for cancer therapy and diagnosis, Yang et al. (2018) incorporated RGD peptide at the surface of manganese oxide (MnO) nanoclusters particles, grafted with a polyethylene glycol layer. Manganese oxide accelerated liberation at U87MG cells would generate more precise cancer images, while the RGD motif can improve the direction of the target, which are the tumor cells. The authors present that RGD targeting of cancer cells that overexpress αvβ3 integrins allowed the selective capture of human glioblastoma U87MG cells [100].

The RGD application in tumor diagnosis was explored by Zheng et al. (2022) in dual-modal magnetic resonance/fluorescent imaging (MRI/FI). The researchers evaluated a dual-modal imaging agent known for its enhanced sensitivity and specificity to improve image quality. They developed a derivative polypeptide-based compound containing RGD groups as integrin-targeting molecules. The RGD motif was attached to gadolinium diethylenetriaminepentaacetic acid (Gd-DTPA) and rhodamine B (RhB), resulting in the derivative RGD-Gd-DTPA-RhB. The authors concluded that this compound has the potential to serve as a contrast agent for tumor targeting. RGD-Gd-DTPA-RhB demonstrated selective tumor uptake and exhibited a high affinity for B16F10 melanoma in mice through its RGD motif. This led to improved imaging results and significant enhancements in both uptake and fluorescent signals compared to the control group [101].

Li et al. (2011) developed RGD-targeted paramagnetic liposomes to improve early tumor detection via magnetic resonance. The RGD was added into lipid bilayers due to their specific bounding to tumor promoted by the interaction with ανβ3-integrin. The integrin ανβ3 is a maker of tumor angiogenesis and its expression is correlated with neo-vessel formation, cell invasion, and tumor migration. The authors concluded that the liposomes presented specific binding to human lung carcinoma cell lines (HUVEC cells) and human umbilical vein endothelial cells (A549 cells). The competition experiments showed that specific interaction was mediated via RGD motif/integrin [102].

The application of RGD into functional biomimetic film sensors to improve cell adhesion, allowing real-time electrochemical detection, was observed by Guo et al. (2012). The authors developed a live cell sensor by covalently bonding RGD-peptide on graphene to detect nitric oxide. Nitric oxide is released from living cells, and its expression is correlated with important biological signaling and also with tumor angiogenesis. The results showed that the motif RGD and its interaction with the extracellular matrix allowed cell attachment, resulting in a high sensitivity and good selectivity of the desired molecule [103].

Using a strategy without the interaction of RGD-integrins, Khaksari et al. (2023) studied the aptamer used for CTC detection. Aptamers are synthetic short and single-stranded DNA or RNA oligonucleotide ligands that form well-defined three-dimensional structures so that they bind to their targets with high specificity and affinity [104,105]. The authors developed an electrochemical microfluidic biosensor for the detection of A549 human adenocarcinoma cells, recognized for being CTCs, through the interaction between α6β4 integrin present on the cell membrane surface and the α6β4 integrin-specific DNA aptamer [30]. The biosensor showed a wide linear dynamic range of 50^−5^ × 10^5^ cells·mL^−1^ and a detection limit of 14 cells·mL^−1^.

### 3.3. EpCAm

The molecule of epithelial cell adhesion—EpCAM, is a transmembrane protein, described as a tumoral prognostic marker and an anchor for circulant tumoral cells highly expressed in carcinomas and their metastasis [106]. EpCAM is a type I glycoprotein transmembrane, consisting of a sequence of 314 amino acids, constituting a large extracellular domain, transmembrane, and cytoplasmic region [107,108]. EpCAM is a mediating molecule for independent Ca^2+^ adhesions, different from typical intercellular junctions like cadherins [109].

The EpCAM superexpression in a tumoral cell is an unknown mechanism. In vitro studies denote that the phenomenon is correlated with the stimulation of the cell cycle, upregulating the proto-oncogene c-myc and inducing the cell proliferation [110].

The upregulated expression of EpCAM denotes a highly aggressive cancer proliferation due to involvement in the regulation of cellular adhesion, migration, proliferation, cycle metabolism, and metastasis, negatively correlating the EpCAM expression with the expected survival of cancer patients. [111]. The literature related that EpCAM expression occurs at a high level of primary carcinoma from the colon, stomach, prostate, lung [112], ovarian, and endometrial cancer [107].

In the literature, many procedures for CTCs detection based on cellular adhesion mediated by EpCAM have been described, however, the sensitivity and the specificity still are a challenge. EpCAM molecules are highly expressed in epithelial cells and cancer, but it is absent in blood cells. Moreover, circulating tumor cells (CTCs) are infrequent, scarce, and prone to genotypic and phenotypic changes [113], making them an attractive target for the development of tumor detection methods.

Chen and co-workers introduced a new microfluidic device fabricated through 3D printing. It exhibited a large surface area and allowed for the manipulation of fluid flow. The device was functionalized with anti-EpCAM antibodies, enabling the capture of circulating tumor cells (CTCs) from peripheral blood samples [114]. The researchers demonstrated a successful capture of EpCAM-positive cancer cell lines, including MCF-7 breast cancer, SW480 colon cancer, and PC3 prostate cancer, with an efficiency exceeding 90%. In the case of the EpCAM-negative cancer cell line (293T kidney cancer), the capture efficiency was measured at 26.14 ± 5.30%.

Ortega et al. (2015) synthesized a microfluidic immunosensor to quantify EpCAM in biological samples. A nanoparticle of silver covered with chitosan (AgNPs-Cts) was functionalized with antibodies anti-EpCAM. The determination of CTCs in peripheral blood occurred with blood samples from patients with metastatic advanced colon cancer, and the detection limit was set at 8 CTCs in 12 mL. The microfluidic immunosensor showed higher sensibility and shorter assay time employed than the common-use, commercial ELISA test, the detection limit was 2.7 pg·mL^−1^ in 34 min, and 13.9 pg·mL^−1^ and 370 min, respectively [115].

Jalil et al. (2021) developed an electrochemical biosensor by immobilizing anti-EpCAM antibodies onto a nanohybrid material consisting of molybdenum disulfide (MoS2) grafted onto reduced graphene oxide (MoS_2_@rGO). The nanohybrid was then electrophoretically deposited onto an indium tin oxide (ITO) coated glass substrate. The effectiveness of the sensor was evaluated using human serum, urine, and saliva samples spiked with 10 ng·mL^−1^ of EpCAM. The results demonstrated that the biosensor exhibited excellent performance in detecting EpCAM in all tested biological mediums, with a recovery rate exceeding 90% [31].

Wu et al. (2023) evaluated CTCs detection in biological systems with an anti-EpCAM functionalized chip aiming to monitor tumor recurrence. 4T1 mouse model of breast cancer was used to evaluate and monitor tumor recurrence. The time necessary to detect CTCs was compared with diagnosis via in vivo bioimaging and pathological examination. The authors described that functionalized chip detected CTCs 10 days before an imaging exam. The study also contributes to reinforcing the concept that CTCs expression has a direct relation with tumorigenesis and metastasis [116].

Hashkavayi and co-workers developed an electrochemical EpCAM aptasensor with a highly selective and sensitive response for CTCs detection using dual signal amplification. The aptasensor was developed immobilizing the EpCAM aptamer in gold nanostructures (GNSTs) and an approach for double signal amplification involving RCA with the catalytic capacity of hemin/G-quadruplex complex. Real sample tests used human serum containing cells of human colon cancer (HT-29) evaluated the recovery potential, resulting in a range of 95–107%. The HT-29 limit of detection was 1 cell·mL^−1^ [113].

Luo et al. (2020) developed a photoelectrochemical biosensor for the detection of CTCs based on a nanocomposite of magnetic nanospheres of Fe_3_O_4_ and anti-EpCAM and a probe of nanoparticles of Cu_2_O and aptamer [60]. The aptamer specifically bound MUC1 overexpressed on the surface of breast cancer tumor cells (MCF-7). In addition, the use of aptamer generated the amplification of the detection response, caused by the greater steric impediment of the working electrode, being an efficient strategy for signal amplification in biosensors. The linear response range was 3 cell·mL^−1^ to 3 × 10^3^ cell·mL^−1^ and a detection limit of 1 cell·mL^−1^. Peng et al. (2022) proposed an electrochemical biosensor controlled by dual recognition, through the interaction between two aptamers and two breast cancer tumor cell surface proteins (MCF-7), mucin 1 (MUC1), and adhesion molecules of epithelial cells (EpCAMs), to improve the accuracy of the device [117]. This had a limit of detection of 3 cells·mL^−1^.

Thus, Table 1 summarizes the main recent publications that address the use of technologies applied to electrochemical biosensing of tumor diseases. These publications deal with the interaction between circulating tumor cells and the biomarkers discussed in this review study.

## 4. Conclusions and Perspectives

Circulating tumor cells (CTCs) are extensively studied for their potential in cancer diagnosis and prognosis. However, there are significant challenges that need to be addressed. The heterogeneity of CTCs poses a difficulty for certain cell adhesion approaches, as they consist of various types such as epithelial tumor cells, epithelial-to-mesenchymal transition cells, hybrid tumor cells, irreversible tumor cells, and circulating tumor stem cells. This heterogeneity makes them undetectable by some established methods [128].

Furthermore, the detection of CTCs is limited by their low abundance in peripheral blood, with approximately only 1 cell present in every 10^5^–10^7^ cells [129]. The biological characteristics of peripheral blood also present a challenge for CTC detection. Cancer-related changes in blood clotting, sedimentation rates, viscosity reduction, and depletion of fibrinogen alter the fluid dynamics of the system, interfering with the capture of CTCs [130].

To overcome these technical challenges, biosensors have shown great promise. They offer high sensitivity and selectivity, low cost, and simplicity [16,23,35]. The development of nanofabrication technologies and microfluidics knowledge has enabled the creation of nanostructured electrodes, which enhance the surface-volume ratio and promote specific interactions between the biorecognition elements and CTCs. Hierarchical topographic structures at the micro and nanoscale mimic the cell environment, facilitating cell adhesion. Studies have demonstrated the effectiveness of such structures in increasing the number of adhered cells compared to flat structures [131,132]. Additionally, the geometry and configuration of these structures impact the performance of biosensors. Recent research has explored the development of 3D electrodes using additive manufacturing, which can improve selectivity and sensitivity properties [32].

Combined methods are used to enhance CTC adhesion and improve cell detection. Aptamer-based methods, utilizing aptamers against specific cancer cell-surface biomarkers (e.g., CD44, Integrins, Ep-CAM), are considered highly promising. These approaches enable specific interactions, are easily developed and modified, detect a variety of targets, and exhibit stability, biocompatibility, and reusability [129,133,134]. Aptamers have been extensively studied for CTC detection and the identification of new cancer biomarkers [63,135,136,137,138]. Moreover, these recognition ligands can amplify the sensor response signal, further enhancing their utility [52,60,61,117,129].

In summary, multidisciplinary efforts are underway to improve the sensitivity and capture of CTCs through the study of sensor coatings, materials, geometries, and technologies. These advancements aim to develop cost-efficient technologies for early tumor diagnosis. Over the past decade, significant progress has been made in understanding cell adhesion mechanisms, but there are still opportunities to explore and investigate, particularly in increasing specificity and target precision. The development of functional and clinically viable sensors holds tremendous potential to revolutionize conventional diagnostic techniques, enhance the quality of life for cancer patients through less invasive methods like liquid biopsy, and improve prognosis and disease progression monitoring, thus increasing chances of successful treatment and recovery.

## Figures and Tables

**Table 1:** Main publications in the last 5 years that address the use of the biomarkers discussed in this article applied to electrochemical biosensors aimed at detecting tumor diseases.

Type of Sensors	Type of Marker	Cancer Cell	Type of Affinity Receptor	Detection Limit	Linear Range	References
Electrochemical immunosensor based on polyamidoamine dendrimer	EpCAM	HepG2	Anti-EpCAM	2.1 × 10^3^ cells·mL^−1^	1 × 10^4^ to 1 × 10^6^ cells·mL^−1^	Xu et al., 2019 [118]
Electrochemical sensor based on carbon nanotube composites and hyaluronic acid and poly(diallyldimethylammnium chloride)	CD44	MCF-7	Hyaluronic acid	660 cells·mL^−1^	-	Zhang et al., 2019 [75]
Sensor based on nanosphere separation and a DNA-generated electrochemical current	EpCAM	MCF-7	Anti-EpCAM	1 cell·mL^−1^	5 to 3 × 10^4^ cells·mL^−1^	Shen et al., 2019 [119]
Electrochemical impedance spectroscopy (EIS) sensor conjugating hyaluronic acid (HA) with bovine serum albumin (BSA)-modified gold nanoparticles (GNPs)	CD44 Receptor	MDA-MB-231, HCT116 and L02	Hyaluronic Acid	128 cells·mL^−1^ for MDA-MB-231 cells, 167 cells·mL^−1^ for HCT116 cells, and 346 cells·mL^−1^ for L02 cells	Range of 2.0 × 10^2^ to 3.0 × 10^5^ cells·mL^−1^ for MDA-MB-231 cells and HCT116 cells, and 5.0 × 10^2^ to 3.0 × 10^5^ cells·mL^−1^ for L02 cells	Zhou et al., 2021 [120]
Electrochemical impedance spectroscopy (EIS) sensor of CD(HA)/TiO_2_/Cu^2+^	CD44 Receptor	MDCK cells	Hyaluronic Acid	2.31 cells·mL^−1^	-	Giang et al., 2021 [121]
Electrochemical sensor based on LiFePO_4_ particles as an electrochemical label	MUC1 protein	MCF-7	Aptamer	1 cell·mL^−1^	3 to 10,000 cells·mL^−1^	Zhang et al., 2020 [122]
Electrochemical sensor using hemin/G-quadruplex complex as a dua- signal amplification strategy	EpCAM	HT-29	Aptamer	1 cell·mL^−1^	5 to 10^7^ cells·mL^−1^	Bagheri Hashkavayi et al., 2021 [113]
Photoelectrochemical platform for sensitive detection of soluble CD44 proteins engineered with MXene-TiO_2_/BiVO_4_ hybrid	CD44 Receptor	CD44	Hyaluronic Acid	1.4 × 10^−2^ pg·mL^−1^	2.2 × 10^−4^ ng·mL^−1^ to 3.2 ng·mL^−1^	Soomro et al., 2020 [123]
Electrochemical sensor using Au/Ti/Si substrate	EpCAM	Capan-2	Aptamer	13 cells·mL^−1^	-	Li et al., 2022 [124]
Electrochemical sensor using Co-Fe-MOF	EpCAM	HepG2, HeLa, MCF7, MDA-MB-468 and MCF-10	Aptamer	11 for HepG2, 9 for HeLa, 10 for MCF7,10 for MB-468, 11 cells·mL^−1^ MCF-10	-	Zhang et al., 2023 [125]
Electrochemical sensor NH_2_-Fe-MOF-Zn nanosheet	CD44	MCF-7	Anti CD44 antibody	-	10^3^ to 10^6^ cells·mL^−1^	Lian et al., 2022 [126]
Electrochemical sensor using BSA/Anti-EGFR/Gold electrode	EpCAM	MCF-7	Aptamer	2 cells·mL^−1^	5 to 1 × 10^6^ cells·mL^−1^	Li et al., 2023 [127]

## Data Availability

Not applicable.
